# Are female scientists underrepresented in self-retractions for honest error?

**DOI:** 10.3389/frma.2023.1064230

**Published:** 2023-01-20

**Authors:** Mariana D. Ribeiro, Jesus Mena-Chalco, Karina de Albuquerque Rocha, Marlise Pedrotti, Patrick Menezes, Sonia M. R. Vasconcelos

**Affiliations:** ^1^Science Education Program, Institute of Medical Biochemistry Leopoldo de Meis (IBqM), Federal University of Rio de Janeiro (UFRJ), Rio de Janeiro, Brazil; ^2^Center for Mathematics, Computing and Cognition (CMCC), Federal University of ABC (UFABC), São Paulo, Brazil

**Keywords:** gender, self-correction of science, retractions, research integrity, research assessment, science policy

## Abstract

Retractions are among the effective measures to strengthen the self-correction of science and the quality of the literature. When it comes to self-retractions for honest errors, exposing one's own failures is not a trivial matter for researchers. However, self-correcting data, results and/or conclusions has increasingly been perceived as a good research practice, although rewarding such practice challenges traditional models of research assessment. In this context, it is timely to investigate who have self-retracted for honest error in terms of country, field, and gender. We show results on these three factors, focusing on gender, as data are scarce on the representation of female scientists in efforts to set the research record straight. We collected 3,822 retraction records, including research articles, review papers, meta-analyses, and letters under the category “error” from the Retraction Watch Database for the 2010–2021 period. We screened the dataset collected for research articles (2,906) and then excluded retractions by publishers, editors, or third parties, and those mentioning any investigation issues. We analyzed the content of each retraction manually to include only those indicating that they were requested by authors and attributed solely to unintended mistakes. We categorized the records according to country, field, and gender, after selecting research articles with a sole corresponding author. Gender was predicted using *Genderize*, at a 90% probability threshold for the final sample (*n* = 281). Our results show that female scientists account for 25% of self-retractions for honest error, with the highest share for women affiliated with US institutions.

## 1. Introduction

Retractions are among the effective measures to strengthen the self-correction of science and thus the reliability and quality of the literature. Concerning self-retractions for honest errors, whereas exposing one's own failures is not a trivial matter for researchers, self-correcting data, results, and/or conclusions for unintended errors has increasingly been perceived as a good research practice (ALLEA, 2017; Global Research Council, 2021; Ribeiro et al., 2022). Recognition for such practice, however, is not (yet) part of the culture of rewards in academia (Bishop, 2018; Nature Human Behavior, 2021). One reason is that mechanisms to correct the literature, with post-publication explanations for invalidating, for example, data and/or conclusions of a research article, including self-retractions for honest error, gained traction only in the last two decades (Fang et al., 2012). Another reason is that those leading science today have built their careers within a culture of rewards based mostly on the publication of research articles and other scientific reports, forming the bedrock of knowledge in most fields, with rare instances of self-correction. In this prevailing culture, “[t]he thought of having to retract an article can instill fear into the heart of scientists, who see it as equivalent to being named and shamed. There are currently few incentives for honesty, and keeping quiet about an error will often seem the easiest option” (Bishop, 2018).

It is thus timely to investigate what factors underlie retractions and self-retractions for honest errors. Previous studies have shown the distribution of retractions and its reasons among journals (Fang and Casadevall, 2011; Fang et al., 2012; Gasparyan et al., 2014; Vuong et al., 2020), research fields (Grieneisen and Zhang, 2012; Ribeiro and Vasconcelos, 2018; Vuong et al., 2020), and countries (Fang et al., 2012; Amos, 2014; Fanelli et al., 2015; Ribeiro and Vasconcelos, 2018). When it comes to reasons for retractions, a considerable share is attributed to misconduct, especially to falsification, fabrication, plagiarism, in different fields, with smaller fractions for honest errors (Fang et al., 2012; Bozzo et al., 2017; Li et al., 2018; Ribeiro and Vasconcelos, 2018; Coudert, 2019; Christopher, 2022). A recent analysis of 330 retractions (2010-2019) in journals indexed in the Web of Science database showed that 66.4% accounted for data results, including falsification, fabrication, and unreliable results (Lievore et al., 2021).

Retractions and self-retractions can reveal much about the social dimension of the scientific enterprise. That said, the understanding of factors influencing this correction process should be sought in light of a research culture, including its publication system, that does not incentivize publicly exposing failures (Allison et al., 2016; Bishop, 2018; Rohrer et al., 2021). When it comes to such exposure through self-retractions for honest error, although there have been growing efforts toward normalizing this process (Bishop, 2018; Ribeiro et al., 2022), such cultural shift takes time. One issue is perceptions among scientists that one's reputation may be tainted in this process (Bishop, 2018; Hosseini et al., 2018). In fact, there are several gaps in our understanding of factors underlying the individual self-correction of science for honest error, including possible influences of fields, countries, and gender. Concerning the latter, given gender disparities in academia, it is worth investigating whether female scientists are more (or less) proactive than male scientists toward correcting the research record for honest errors, across fields and countries. As well documented, gender disparities are part of the history of science, and they have posed several barriers that female scientists have to overcome for being recognized in academia.

An American perspective on this matter was brought by Margaret Rossiter, a well-known science historian who coined the phrase “*Matilda Effect*” (Rossiter, 1993). Different from the Matthew Effect, a biased recognition toward those who are already eminent in science (Merton, 1968), the Matilda Effect is the result of prejudice that women face in academia, leading their work to be overlooked or even credited to male colleagues (Rossiter, 1993; Lincoln et al., 2012). Female researchers themselves may have implicit gender biases against their own peers (Lincoln et al., 2012; Knobloch-Westerwick et al., 2013; Raymond, 2013).

That said, gender inequalities remain a challenge for women in science. For example, a comprehensive study on gender disparities in science across 83 countries and 13 disciplines shows that the gender gap in terms of research productivity is a widespread phenomenon (Huang et al., 2020). Female scientists secure fewer first and last authorship positions (Larivière et al., 2013; West et al., 2013; Hart and Perlis, 2019; Ross et al., 2022), compound fewer peer-review and editorial boards (Helmer et al., 2017), tend to publish in lower impact journals in some fields (Larivière et al., 2013; Bendels et al., 2018; Molwitz et al., 2021), usually receive fewer citations (Larivière et al., 2013; Bendels et al., 2018; Shamsi et al., 2022), less funding (Ley and Hamilton, 2008; Oliveira et al., 2019), fewer awards (Lincoln et al., 2012; Meho, 2021), and patents (Ross et al., 2022).

It is against this backdrop that we have explored the role of gender in self-correcting the research record. For example, this unfavorable environment for women in academia may make female authors more reluctant to self-retract research articles, even for honest errors, as they might fear the outcome. Looking at gender and retractions, Decullier and Maisonneuve (2021) found that among 120 retractions analyzed, 37.2% were authored by female authors, with male authors accounting for 59.2% for fraud and plagiarism. However, this analysis was not based on sole corresponding authors, who are expected to have a decisive role in initiating a retraction. We explored the representation of gender in self-correcting science through research articles with sole corresponding authors, based on a dataset of 3,822 retractions attributed to error.

## 2. Methodology

We collected data on retractions classified under the category *error* from The Retraction Watch Database (2018) (01/01/2010–12/31/2021 – 12 years in total). A total of 3,822 records were obtained, with information on authorship, article type, DOI of the original publication, DOI of the retraction notice, and nature of the publication: research article, letter, case report, review article, clinical study, conference abstract, meta-analysis, preprint, commentary/editorial, book chapter, auto/biography, trade magazine, correction/erratum, guideline, governmental publication, interview, supplementary material.

We selected only research articles (RA), considering the impact of the correction of original data on the research record. After excluding any record that mentioned “investigation by” as such categorization may involve other reasons rather than honest error, we screened the dataset for retraction for error in analyses and/or data and/or methods and/or materials and/or conclusions and/or image and/or text. We excluded records that combined this information with at least one of the following: false/forged authorship, paper mill, ethical violations, and/or fake peer review, misconduct, falsification, fabrication, plagiarism, publisher or third party, concerns/issues about data or original data not provided (when retraction notice was not clear), concerns/issues about data and/or authorship and/or referencing/attributions, duplication, manipulation. This refinement was necessary to prevent that error and misconduct, or error and other unknown or even obscure reasons would be categorized as honest error. We collected the ISSN of each journal, of each original paper, with category information, Impact Factor (IF), field, according to Journal Citation Reports [JCR, with information made available by Clarivate Analytics (2022)], and country. For the RAs, those with unidentified or with more than one corresponding author were excluded. We obtained 575 notices and excluded those with insufficient information in our search (*n* = 11), with unclear or obscure reasons, with an indication that the retraction was not initiated by the authors, and with more than one corresponding author (“raw dataset”). We obtained 472 self-retractions with only one corresponding author, initially attributed to honest error. This final dataset (*n* = 472) included the complete names (at least first name and surname) of the corresponding authors. These names were determined manually, by comparing information from publications, institutional and personal websites, affiliations, and e-mail addresses.

**Figure 1:**
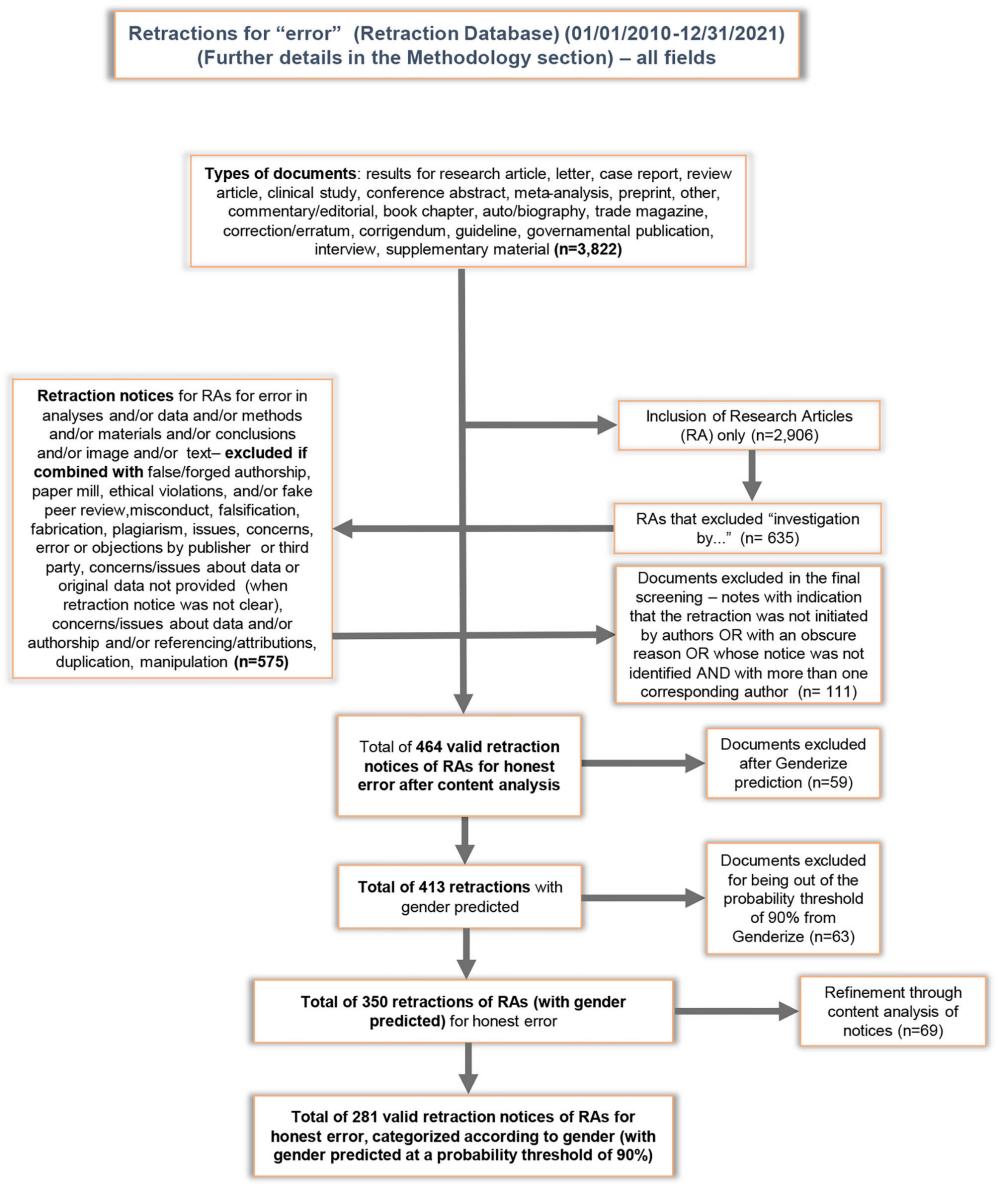
Screening scheme for the final dataset on self-retractions for honest error of research articles with sole corresponding authors (*n* = 464), with gender predicted at a probability threshold of 90% (*n* = 281), originally extracted from 3,822 records for retractions for error in The Retraction Watch Database (2018) for the period 2010–2021.

Concerning gender assignment, we used the Genderize database for gender prediction, which is based on total counts of a first name and on the probability of prediction. Further details can be found at https://genderize.io. The gender of these corresponding authors was predicted for 413 (88%) of the 472 corresponding authors included in our refined dataset. After a preliminary analysis of the content of each notice in the “raw dataset” (*n* = 564), a total of 464 self-retractions for honest error were validated by two members of the team. From the 413 with gender predicted, a total of 350 was obtained after further refinement, but notices with gender prediction below 90% were excluded (*n* = 69). A total of 281 valid self-retraction notices attributed to honest error were obtained and classified according to gender. Figure 1 shows the steps of our screening scheme.

## 3. Results and discussion

We selected all retraction notices, classified under the category *error*, of research articles from the period 2010 and 2021, collected from The Retraction Watch Database (2018), and set up a dataset with 3,822 retraction records for error. As shown in the screening scheme in Figure 1, we obtained 281 self- retractions for honest error of research articles with only one corresponding author, with gender predicted with a 90% probability threshold. This number is equivalent to 61% of the total self-retractions of research articles with notices exclusively attributed to honest error (*n* = 464) in our dataset, including those research articles that had more than one corresponding author. This number (*n* = 464) is equivalent to 16% of the 2,906 retractions of research articles classified under the category of “error” in the Retraction Watch Database for the period 2010–2021.

**Figure 2:**
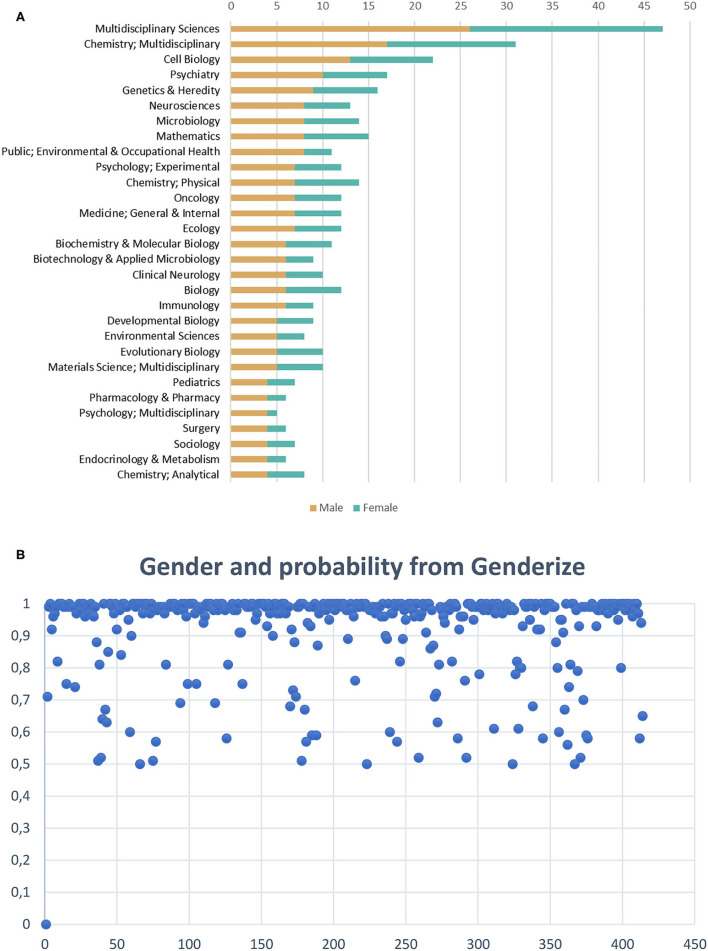
**(A)** Distribution across fields of corresponding authors in terms of gender (female vs. male) of valid self-retractions for honest error of research articles (*n* = 281) recorded in the Retraction Watch Database (2010–2021), with female corresponding authors accounting for 25% of these records. The data are plotted for the 30 most frequent categories. **(B)** Distribution of prediction probability (between 50 and 100%) for female and male names of corresponding authors of research articles self-retracted (*n* = 413), predicted by Genderize.

Figure 2A shows the distribution of valid self-retractions for honest error of research articles (*n* = 281, with only one corresponding author) across fields, according to Journal Citation Reports (JCR), with gender predicted by Genderize, with 25% female- and 75% male-authored records. Figure 2B offers an overview of the distribution of probability for the name of the corresponding being female or male.

The results show that 25% (*n* = 71) of valid self-retraction notices of research articles for error (*n* = 281) (2010–2021) were led by female scientists, who were the sole corresponding authors of the research articles. A previous study exploring characteristics, global distributions, and reasons for 1,339 retractions from PubMed and Retraction Watch website showed that “[f]or all reasons of retraction, the percentage of retracted articles with male senior or corresponding authors was substantially higher than that with female senior or corresponding authors” (Li et al., 2018, p. 41). As for retractions for error, the same authors reported that female corresponding authors accounted for 19.2% (*n* = 37) of the total of retractions attributed to error (*n* = 193) (Li et al., 2018). Decullier and Maisonneuve (2021) investigated the underrepresentation of women in retractions and identified the reasons for 113 retractions for female and male authors and found that 37.2% retractions were for publications first authored by female scientists. The study also showed that reasons for retraction differed considerably comparing female and male authors, with 28.6% of retractions for research misconduct for female and 59.2% for male authors (Decullier and Maisonneuve, 2021). These percentages are consistent with evidence brought by Fang et al. (2013), who revealed that male scientists were overrepresented (about two thirds of 228 individuals) among those committing research misconduct in the life sciences.

Drawing upon Nosek et al. (2007) and Pohlhaus et al. (2011), Kaatz et al. (2013) reported that “[i]f we use NIH research award dollars as a proxy for the opportunity to commit fraud in the life sciences, we find that men have substantially more opportunity to commit fraud than women. Compared to women, men are more likely to hold multiple simultaneous R01 awards, lead large center grants, and successfully compete when submitting renewals (20–22)” (Kaatz et al., 2013; p. 2).

**Figure 3:**
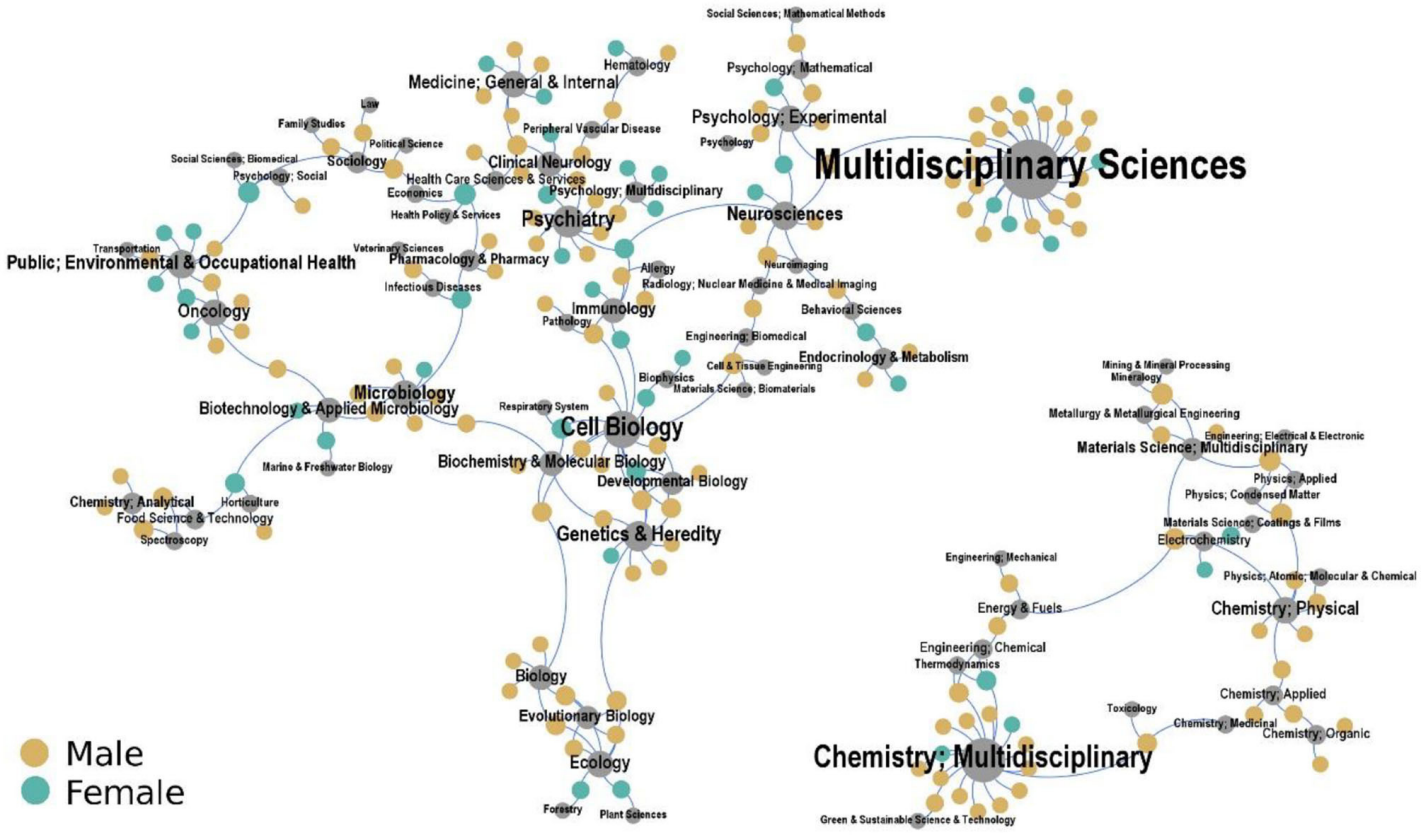
Network of self-retractions for honest error, considering research articles (*n* = 244), across fields (according to JCR), recorded in the Retraction Watch Database (2010–2021) and combined with information from the Web of Science, for sole corresponding authors, female or male. Note that the fields for 37 documents in our final dataset were not identified.

This overrepresentation of male scientists is also marked in our dataset, across fields (Figure 3). In this figure, each corresponding author is associated to the article field (gray vertex). The size of each vertex is proportional to the number of authors associated with it. In order to simplify the data visualization, Figure 3 displays only the two largest connected components of the generated network.

Figure 3 shows self-retractions for honest error by male corresponding authors, compared to female authors, distributed across the fields. As can be seen, male scientists are prevalent for most of these fields, including multidisciplinary and chemistry multidisciplinary (83%, with 34 out 41 records for these fields), and the medical, biomedical, life (including environmental) and health sciences (67%, with 96 out 143 records for these fields). The prevalence of self-retractions of male corresponding authors in these latter fields might suggest that they are more proactive than female corresponding authors toward this type of correction. Nevertheless, the high-impact-factor of most journals (Table 1) may be a confounder. Note, for example, that male corresponding authors account for 82% (*n* = 46) of 56 research articles with valid self-retractions for honest error, published in journals with impact factors between 10 and 176.

**Table 1:** List of 46 research articles (82%), out a total of 56, with valid self-retractions for honest error authored by male scientists, published in journals with impact factors between 10 and 176, according to JCR.

**DOI**	**Fields according to journal citation reports (JCR)**	**Impact factor (IF)**
10.1161/STROKEAHA.120.031889	Peripheral Vascular Disease - Scie(Q1); Clinical Neurology - Scie(Q1)	10.2
10.1161/ATVBAHA.119.312964	Hematology - Scie(Q1); Peripheral Vascular Disease - Scie(Q1)	10.5
10.1016/j.scitotenv.2019.04.081	Environmental Sciences - Scie(Q1)	10.8
10.1016/j.cub.2013.11.061	Cell Biology - Scie(Q1); Biochemistry and Molecular Biology - Scie(Q1); Biology - Scie(Q1)	10.9
10.1039/C3GC40855H	Chemistry, Multidisciplinary - Scie(Q1); Green and Sustainable Science and Technology - Scie(Q1)	11
10.1016/j.ajhg.2017.09.002	Genetics and Heredity - Scie(Q1)	11
10.1016/j.psychres.2017.01.028	Psychiatry - Ssci(Q1); Psychiatry - Scie(Q1)	11.2
10.1021/acs.est.0c01643	Environmental Sciences - Scie(Q1); Engineering, Environmental - Scie(Q1)	11.4
10.1212/WNL.0b013e3181ec7fbd	Clinical Neurology - Scie(Q1)	11.8
10.1093/jnci/djq427	Oncology - Scie(Q1)	11.8
10.1038/cdd.2008.151	Biochemistry and Molecular Biology - Scie(Q1); Cell Biology - Scie(Q1)	12.1
10.1073/pnas.1006608107	Multidisciplinary Sciences - Scie(Q1)	12.8
10.1073/pnas.1404082111	Multidisciplinary Sciences - Scie(Q1)	12.8
10.1073/pnas.1613440113	Multidisciplinary Sciences - Scie(Q1)	12.8
10.1073/pnas.0709477105	Multidisciplinary Sciences - Scie(Q1)	12.8
10.1073/pnas.0904984106	Multidisciplinary Sciences - Scie(Q1)	12.8
10.1073/pnas.0608599104	Multidisciplinary Sciences - Scie(Q1)	12.8
10.1101/gad.1832709	Genetics and Heredity - Scie(Q1); Developmental Biology - Scie(Q1); Cell Biology - Scie(Q1)	12.9
10.1101/gad.2004211	Genetics and Heredity - Scie(Q1); Developmental Biology - Scie(Q1); Cell Biology - Scie(Q1)	12.9
10.1111/anae.14939	Anesthesiology - Scie(Q1)	12.9
10.1016/j.devcel.2008.11.006	Cell Biology - Scie(Q1); Developmental Biology - Scie(Q1)	13.4
10.1021/acscatal.6b03687	Chemistry, Physical - Scie(Q1)	13.7
10.1016/j.ophtha.2018.03.003	Ophthalmology - Scie(Q1)	14.3
10.1016/j.jaci.2015.12.1312	Immunology - Scie(Q1); Allergy - Scie(Q1)	14.3
10.1016/j.jaci.2014.12.013	Immunology - Scie(Q1); Allergy - Scie(Q1)	14.3
10.1021/ja807685r	Chemistry, Multidisciplinary - Scie(Q1)	16.4
10.1021/ja501979g	Chemistry, Multidisciplinary - Scie(Q1)	16.4
10.1002/anie.201400623	Chemistry, Multidisciplinary - Scie(Q1)	16.8
10.1002/anie.201704704	Chemistry, Multidisciplinary - Scie(Q1)	16.8
10.1038/s41559-020-01306-x	Ecology - Scie(Q1); Evolutionary Biology - Scie(Q1)	19.1
10.1038/s41562-017-0132	Multidisciplinary Sciences - Scie(Q1); Psychology, Experimental - Ssci(Q1); Neurosciences - Scie(Q1)	24.3
10.1038/nchem.2320	Chemistry, Multidisciplinary - Scie(Q1)	24.3
10.1038/nchem.2551	Chemistry, Multidisciplinary - Scie(Q1)	24.3
10.1182/blood-2015-10-672766	Hematology - Scie(Q1)	25.5
10.1001/jamapsychiatry.2014.1193	Psychiatry - Scie(Q1); Psychiatry - Ssci(Q1)	25.9
10.1001/jamapediatrics.2018.1012	Pediatrics - Scie(Q1)	26.8
10.1136/annrheumdis-2017-211351	Rheumatology - Scie(Q1)	28
10.1001/jamaoncol.2018.3054	Oncology - Scie(Q1)	33
10.1016/S2213-8587(17)30366-2	Endocrinology and Metabolism - Scie(Q1)	44.9
10.1126/science.1104696	Multidisciplinary Sciences - Scie(Q1)	63.7
10.1126/science.1210400	Multidisciplinary Sciences - Scie(Q1)	63.7
10.1126/science.1125731	Multidisciplinary Sciences - Scie(Q1)	63.7
10.1126/science.1259464	Multidisciplinary Sciences - Scie(Q1)	63.7
10.1001/jama.2016.20036	Medicine, General and Internal - Scie(Q1)	157.3
10.1056/NEJMoa1712231	Medicine, General and Internal - Scie(Q1)	176.1
10.1056/NEJMoa1200303	Medicine, General and Internal - Scie(Q1)	176.1

High-impact factor journals would tend to correct more (Fang and Casadevall, 2011; Fang et al., 2012; Brainard, 2018) than the other journals. Additionally, the fact that the medical, biomedical, life, and health sciences have taken the lead in the discussion of publication ethics is likely to influence this pattern.

**Figure 4:**
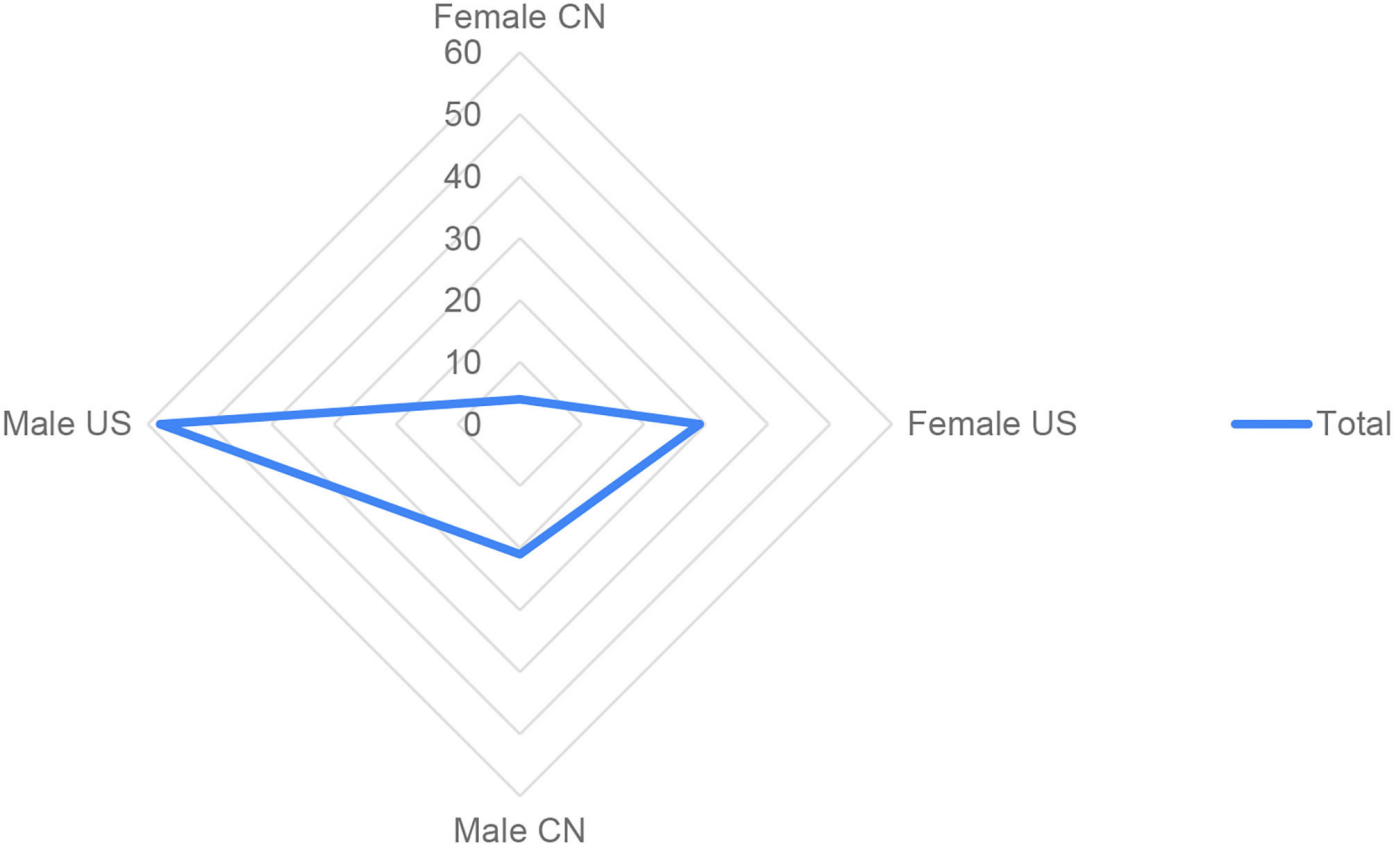
Distribution of self-retractions for honest error (*n* = 112) of research articles recorded in the Retraction Watch Database (2010–2021), for sole corresponding authors affiliated with institutions from the United States (*n* = 87) and China (*n* = 25), with 33% (*n* = 29), and 16% (*n* = 4) female-authored notices.

Overall, and in accordance with previous works already cited in this section, our results indicate that self-retractions for honest error are mostly male led. We categorized the retractions for honest error in our dataset according to country and found that 87 records (31% of the total of 281) were from sole corresponding authors affiliated with institutions in the United States. The country accounts for 41% of all female scientists (*n* = 71) in our dataset with gender predicted (*n* = 281) (Figure 4).

On the one hand, these data do not allow us to infer that female scientists affiliated with the two most productive countries in terms of publication output (National Science Foundation, 2021) have taken a more proactive role in self-correcting science for honest error. In addition to the small size of our sample, there might be “false positives” – for example, some of these corresponding authors who retracted the paper for honest error could have done that not by their own initiative but by a request from editors or a third party. On the other hand, and despite these caveats, these results are at least intriguing. Considering that the United States is one of the major countries leading discussions and actions toward addressing gender disparities in science in the last decades, these data might be interpreted as reflecting this factor. Concerning China, although our data are limited, it is interesting to note that gender disparities continue to be challenging for Chinese scientists, although Gu (2021) suggests that great strides have been taken in the last decades.

As gender biases and disparities have been increasingly recognized as sources of damage for the career of researchers and for the research enterprise at large, in many countries and fields, these results add another layer to the growing body of literature addressing the influence of gender issues in the publication system for female researchers, across countries. In this publication arena, Rohrer et al. (2021), p. 1,265; note that “researchers may often be reluctant to initiate a retraction given that retractions occur most commonly as a result of scientific misconduct (Fang et al., 2012) and are, therefore, often associated in the public imagination with cases of deliberate fraud.” Also, we believe the “potentially high perceived cost of public self-correction” (Rohrer et al., 2021) might reflect on the attitude of female scientists toward errors in data, results, and conclusions in their research articles. Given the well-known gender issues in academia, female corresponding authors may have mixed feelings about self-correcting the literature, even for honest errors.

## 4. Conclusions

Our results indicate that the percentage of self-retractions that can be attributed solely to unintended mistakes in research articles is low, compared to other reasons, at least for this 12-year period. As we have shown, this category (honest error) accounts for only 16% of our raw sample of 2,906 retraction notices. When it comes to gender, we have found that self-retractions for honest error have been mostly male led, with prevalence for corresponding authors affiliated with institutions in the United States and China in our sample. According to our results, male corresponding authors account for 75% of the notices, which might reflect that gender disparity trickles down to retractions, corroborating previous results, and to self-reporting errors for research papers.

Perhaps one possible explanation for this finding may be that these male corresponding authors have come across post-publication issues, for example, in their data, results, and/or conclusions, more often than female corresponding authors. However, these possible explanations cannot tell the whole story, considering the social dimension of retractions. This apparent underrepresentation of these female scientists merits further investigation.

As we had suggested previously, the perception that retractions would taint the reputation of scientists might be stronger among women, which may be a source of unconscious bias in this correction process. After all, “[e]xisting recognition and reward structures offer no external incentive to come forward and request a retraction of your paper upon discovering a fatal honest error.” (Nature Human Behavior, 2021, p. 1,591). As social structures in academia are entangled with gender disparities, whether such disparities have played a role in discouraging female scientists, at different career stages, to come forward and correct the research record for honest error through self-retractions is a wide-open question.

## 5. Limitations

Our study has several limitations worth noting. First, the source of the data are subject to research material that is not free from bias — the Retraction Watch Database records information from retraction notices whose content is not necessarily detailed and may involve overlapping classifications. For example, not everything classified under the category error is restricted to it as retractions can include error and other issues not always detailed by editors and/or authors. That said, our category “honest error”, although resulting from a careful screening and independent crosschecking of the notices, relies mostly on the honesty of the authors. Whereas we applied stringent criteria to include honest-error notices in our sample, we cannot take for granted that all these notices are overly honest and/or bias-free reports.

Second, we adopted a binary (female or male) category for gender, which is the only possible given restrictions imposed by the way the publication system is organized so far. The third issue is the threshold used for gender prediction, obtained from Genderize, which is conservative, as of 90%. Yet, a less conservative threshold — starting at 75%, for example, leads to an increment of only 4% in the representation of female corresponding authors. One additional caveat is the size of our sample of valid self-retractions for honest error with reliable gender prediction (*n* = 281), equivalent to 61% of the 464 valid self-retraction notices for honest error obtained.

Despite this caveat, we set up strict criteria for a notice to be considered a self-retraction and exclusively attributed to error. We thus believe, on the basis of such criteria, that our results offer a reliable picture of the representation of gender (female vs. male) in the self-correction of science for honest error through retractions. Considering the crucial role of corresponding authors to help correct the research record, further studies exploring the representation of female scientists in this process are timely.

## Data availability statement

The raw data supporting the conclusions of this article will be made available by the authors, on request, without undue reservation.

## Author contributions

MR, JM-C, KR, MP, PM, and SV: substantial contributions to the conception, design of the work, the acquisition, analysis, interpretation of data for the work, drafting the work, revising it critically for important intellectual content, final approval of the version to be published, and agreement to be accountable for all aspects of the work. SV, MR, MP, and JM-C: final revision. All authors contributed to the article and approved the submitted version.

## References

[B1] ALLEA (2017). All European Academies. The European Code of Conduct for Research Integrity. Berlin: All European Academies. Available online at: https://www.allea.org/wp-content/uploads/2017/05/ALLEA-European-Code-of-Conduct-for-Research-Integrity-2017.pdf (accessed June 16, 2022).

[B2] Allison D. B. Brown A. W. George B. J. Kaiser K. A. (2016). Reproducibility: A tragedy of errors. Nature 530, 27–29. 10.1038/530027a 26842041 PMC4831566

[B3] Amos K. (2014). The ethics of scholarly publishing: exploring differences in plagiarism and duplicate publication across nations. J. Med. Libr. Assoc. 102, 87–91. 10.3163/1536-5050.102.2.005 24860263 PMC3988779

[B4] Bendels M. H. K. Müller R. Brueggmann D. Groneberg D. A. (2018). Gender disparities in high-quality research revealed by Nature Index journals. PLoS ONE 13, e0189136. 10.1371/journal.pone.0189136 29293499 PMC5749692

[B5] Bishop D. V. M. (2018). Fallibility in science: Responding to errors in the work of oneself and others. Adv. Methods Pract. Psychol. Sci. 1, 432–438. 10.1177/2515245918776632

[B6] Bozzo A. Bali K. Evaniew N. Ghert M. (2017). Retractions in cancer research: a systematic survey. Res. Integr. Peer Rev. 2, 1–7. 10.1186/s41073-017-0031-1 29451549 PMC5803635

[B7] Brainard J. (2018). Rethinking retractions. Science 362, 390–393. 10.1126/science.362.6413.390 30361352

[B8] Christopher M. M. (2022). Comprehensive analysis of retracted journal articles in the field of veterinary medicine and animal health. BMC Vet. Res. 18, 1–15. 10.1186/s12917-022-03167-x 35180878 PMC8855588

[B9] Clarivate Analytics. (2022). Journal Citation Reports. Available online at: https://clarivate.com/webofsciencegroup/solutions/journal-citation-reports/ (accessed August 24, 2022).

[B10] Coudert F. X. (2019). Correcting the scientific record: retraction practices in chemistry and materials science. Chem. Mater. 31, 3593–3598. 10.1021/acs.chemmater.9b00897

[B11] Decullier E. Maisonneuve H. (2021). Retraction according to gender: A descriptive study. Account Res. 1–6. 10.1080/08989621.2021.1988576. [Epub ahead of print]. 34615410

[B12] Fanelli D. Costas R. Larivière V. (2015). Misconduct policies, academic culture and career stage, not gender or pressures to publish, affect scientific integrity. PLoS ONE 10, e0127556. 10.1371/journal.pone.0127556 26083381 PMC4471332

[B13] Fang F. Casadevall A. (2011). Retracted science and the retraction index. Infect. Immun. 79, 3855–3859. 10.1128/IAI.05661-11 21825063 PMC3187237

[B14] Fang F. Steen R. Casadevall A. (2012). Misconduct accounts for the majority of retracted scientific publications. PNAS 109, 17028–17033. 10.1073/pnas.1212247109 23027971 PMC3479492

[B15] Fang F. C. Bennett J. W. Casadevall A. (2013). Males are overrepresented among life science researchers committing scientific misconduct. mBio 4, e00640–e00612. 10.1128/mBio.00640-12 23341553 PMC3551552

[B16] Gasparyan A. Ayvazyan L. Akazhanov N. Kitas G. (2014). Self-correction in biomedical publications and the scientific impact. Croat. Med. J. 55, 61–72. 10.3325/cmj.2014.55.61 24577829 PMC3944419

[B17] Global Research Council (2021). Responsible Research Assessment. UK, 1-28. Available online at: https://globalresearchcouncil.org/fileadmin/documents/GRC_Publications/GRC_RRA_Conference_Summary_Report.pdf

[B18] Grieneisen M. Zhang M. (2012). A comprehensive survey of retracted articles from the scholarly literature. PLoS ONE 7, e44118. 10.1371/journal.pone.0044118 23115617 PMC3480361

[B19] Gu C. (2021). Women scientists in China: current status and aspirations, *Natl. Sci. Rev*. 8, nwab101. 10.1093/nsr/nwab101 34858614 PMC8566168

[B20] Hart K. L. Perlis R. H. (2019). Trends in proportion of women as authors of medical journal articles, 2008-2018. JAMA Intern. Med. 179, 1285–1287. 10.1001/jamainternmed.2019.0907 31135815 PMC6547141

[B21] Helmer M. Schottdorf M. Neef A. Battaglia D. (2017). Gender bias in scholarly peer review. eLife 6, e21718. 10.7554/eLife.21718.012 28322725 PMC5360442

[B22] Hosseini M. Hilhorst M. de Beaufort I. Fanelli D. (2018). Doing the right thing: a qualitative investigation of retractions due to unintentional error. Sci. Eng. Ethics 24,189–206. 10.1007/s11948-017-9894-2 28321689

[B23] Huang J. Gates A. J. Sinatra R. Barabási A. L. (2020). Historical comparison of gender inequality in scientific careers across countries and disciplines. PNAS 117, 4609–4616. 10.1073/pnas.1914221117 32071248 PMC7060730

[B24] Kaatz A. Vogelman P. N. Carnes M. (2013). Are men more likely than women to commit scientific misconduct? Maybe, maybe not. mBio 4, e00156–e00113. 10.1128/mBio.00156-13 23532977 PMC3622921

[B25] Knobloch-Westerwick S. Glynn C. Huge M. (2013). The Matilda Effect in science communication. Sci. Commun. 35, 603–625. 10.1177/1075547012472684

[B26] Larivière V. Ni C. Gingras Y. Cronin B. Sugimoto C. (2013). Bibliometrics: Global gender disparities in science. Nature 504, 211–213. 10.1038/504211a 24350369

[B27] Ley T. J. Hamilton B. H. (2008). Sociology. The gender gap in NIH grant applications. Science 322, 1472–1474. 10.1126/science.1165878 19056961

[B28] Li G. Kamel M. Jin Y. Xu M. K. Mbuagbaw L. Samaan Z.. (2018). Exploring the characteristics, global distribution and reasons for retraction of published articles involving human research participants: a literature survey. J. Multidiscip. Healthc. 11, 39–47. 10.2147/JMDH.S151745 29403283 PMC5779311

[B29] Lievore C. Rubbo P. dos Santos C. B.. (2021). Research ethics: a profile of retractions from world class universities. Scientometrics 126, 6871–6889. 10.1007/s11192-021-03987-y 34054160 PMC8141102

[B30] Lincoln A. Pincus S. Koster J. Leboy P. (2012). The Matilda Effect in science: Awards and prizes in the US, 1990s and 2000s. Soc. Stud. Sci. 42, 307–320. 10.1177/0306312711435830 22849001

[B31] Meho L. I. (2021). The gender gap in highly prestigious international research awards, 2001–2020. Quant. Sci. Stud. 2, 976–989. 10.1162/qss_a_00148

[B32] Merton R. K. (1968). The Matthew effect in science: the reward and communication systems of science are considered. Science 159, 56–63. 10.1126/science.159.3810.56 5634379

[B33] Molwitz I. Yamamura J. Ozga A. K. Wedekind I, Nguyen, T. Wolf L.. (2021). Gender trends in authorships and publication impact in Academic Radiology—a 10-year perspective. Eur. Radiol. 31, 8887–8896. 10.1007/s00330-021-07928-4 34009412 PMC8589752

[B34] National Science Foundation (2021). Publications output: U.S. Trends and international comparisons. https://ncses.nsf.gov/pubs/nsb20214 (accessed September 9, 2022).

[B35] Nature Human Behavior (2021). Breaking the stigma of retraction. Nat. Hum. Behav. 5, 1591. 10.1038/s41562-021-01266-7 34916652

[B36] Nosek B. A. Smyth F. L. Hansen J. J. Devos T. Lindner N. M. Ranganath K. A.. (2007). Pervasiveness and correlates of implicit attitudes and stereotypes. Eur. Rev. Soc. Psychol. 18, 36–88. 10.1080/10463280701489053

[B37] Oliveira D. F. M. Ma Y. Woodruff T. K. Uzzi B. (2019). Comparison of national institutes of health grant amounts to first-time male and female principal investigators. JAMA 321, 898–900. 10.1001/jama.2018.21944 30835300 PMC6439593

[B38] Pohlhaus J. R. Jiang H. Wagner R. M. Schaffer W. T. Pinn V. W. (2011). Sex differences in application, success, and funding rates for NIH extramural programs. Acad. Med. 86, 759–767. 10.1097/ACM.0b013e31821836ff 21512358 PMC3379556

[B39] Raymond J. (2013). Most of us are biased. Nature 495, 33–34. 10.1038/495033a 23467152

[B40] Ribeiro M. Vasconcelos S. (2018). Retractions covered by Retraction Watch in the 2013–2015 period: prevalence for the most productive countries. Scientometrics 114, 719–734. 10.1007/s11192-017-2621-6

[B41] Ribeiro M. D. Kalichman M. Vasconcelos S. M. R. (2022). Scientists should get credit for correcting the literature. Nat. Hum. Behav. 10.1038/s41562-022-01415-6. [Epub ahead of print]. 35798887

[B42] Rohrer J. M. Tierney W. Uhlmann E. L. DeBruine L. M. Heyman T. Jones B.. (2021). Putting the self in self-correction: Findings from the Loss-of-Confidence Project. Perspect. Psychol. Sci. 16, 1255–1269. 10.1177/1745691620964106 33645334 PMC8564260

[B43] Ross M. Glennon B. Murciano-Goroff R. Berkes E. Weinberg B. Lane J. (2022). Women are credited less in science than men. Nature 608, 135–145. 10.1038/s41586-022-04966-w 35732238 PMC9352587

[B44] Rossiter M. (1993). The Matthew Matilda Effect in science. Soc. Stud. Sci. 23, 325–341. 10.1177/030631293023002004

[B45] Shamsi A. Lund B. Mansourzadeh M. J. (2022). Gender disparities among highly cited researchers in biomedicine, 2014-2020. JAMA Netw. Open 5, e2142513. 10.1001/jamanetworkopen.2021.42513 34994797 PMC8742200

[B46] The Retraction Watch Database. (2018). New York, NY: The Center for Scientific Integrity. Available online at: https://retractionwatch.com/retraction-watch-database-user-guide/ (accessed July 12, 2022).

[B47] Vuong Q. La V. Ho M. Vuong T. Ho M. (2020). Characteristics of retracted articles based on retraction data from online sources through February 2019. Sci. Ed. 7, 34–44. 10.6087/kcse.187

[B48] West J. D. Jacquet J. King M. M. Correll S. J. Bergstrom C. T. (2013). The role of gender in scholarly authorship. PLoS ONE 8, e66212. 10.1371/journal.pone.0066212 23894278 PMC3718784

